# Preventable admissions and emergency-department-visits in pediatric asylum-seeking and non-asylum-seeking patients

**DOI:** 10.1186/s12939-020-01172-w

**Published:** 2020-05-01

**Authors:** Julia Brandenberger, Kayvan Bozorgmehr, Florian Vogt, Thorkild Tylleskär, Nicole Ritz

**Affiliations:** 1 University of Basel Children’s Hospital, Migrant Health Service, Basel, Switzerland; 2 Pediatric Emergency Department, University Children’s Hospital, Bern, Switzerland; 3 Department of General Practice and Health Services Research, University Hospital Heidelberg, Heidelberg, Germany; 4 Department of Population Medicine and Health Services Research School of Public Health, Bielefeld University, Bielefeld, Germany; 5 Unit of NTDs, Department of Clinical Sciences, Institute of Tropical Medicine, Antwerp, Belgium; 6 Centre for International Health, University of Bergen, Bergen, Norway; 7 University of Basel Children’s Hospital, Pediatric Infectious Disease and Vaccinology, Basel, Switzerland; 8 Department of Pediatrics, Royal Children’s Hospital Melbourne, University of Melbourne, Melbourne, Australia

**Keywords:** Migrant health, Refugee health, Immigrant children, Use of health care, Ambulatory-care-sensitive conditions, Emergency department, Health care delivery, Equitable access to health care

## Abstract

**Background:**

Migrant health has become an essential part of public health. According to the World Health Organization, many health systems in Europe have not yet adapted adequately to the needs of asylum-seekers, which might result in untimely and inefficient health care for asylum-seeking patients. The aim of this study was to assess the number of preventable hospital admissions and emergency department visits in asylum-seeking and non-asylum-seeking pediatric patients.

**Methods:**

This is a retrospective, hospital-based study. The study was done at the University Children’s Hospital Basel in Switzerland. Patients admitted or presenting to the emergency department were included and split into the groups of asylum-seeking and non-asylum-seeking patients.

All admissions and emergency-department visits were extracted from the administrative electronic health records from 1st Jan 2016-31st Dec 2017. The main outcome was the proportion of admissions due to ambulatory-care-sensitive conditions (which refer to conditions for which admission can be prevented by early interventions in primary care) in asylum-seeking and non-asylum-seeking patients. Ambulatory-care-sensitive conditions were defined by a validated list of ICD-10 codes.

The secondary objective was to assess the number of preventable emergency-department visits by asylum-seeking patients defined as proportion of visits with a non-urgent triage score.

**Results:**

A total of 75′199 hospital visits were included, of which 63′405 were emergency department visits and 11′794 were admissions. Ambulatory-care-sensitive conditions accounted for 12.1% (18/149) of asylum-seeking and 10.9% (1270/11645) of non-asylum seeking patients’ admissions. Among the emergency department visits by asylum-seeking patients, non-urgent conditions accounted for 82.2% (244/297).

**Conclusions:**

Admissions due to ambulatory-care-sensitive conditions are comparable in asylum-seeking and non-asylum-seeking children, suggesting few delayed presentations to ambulatory care facilities. Strategies to prevent non-urgent visits at pediatric emergency department facilities are needed.

## Background

Migrant health has become an essential part of public health, with one billion people on the move globally [1, 2]. Health systems in Europe may require adaptation for the needs of migrants and asylum-seekers and according to the World Health Organization, many health systems in Europe have not yet adapted adequately [3, 4]. For effective health care, it is essential that everyone – including asylum-seekers – has timely access to the required level of care [5–7]. Asylum-seekers may have an increased risk of delayed or restricted access to health care and consequently a protracted disease course [8, 9]. This can result from a reluctance to access the health care system by asylum-seekers or the absence of knowledge on ways to seek for medical help [7, 10, 11]. Asylum-seeking pediatric patients are a vulnerable group and their health status may be additionally affected by limited access to health care before leaving the country of origin and detrimental conditions during the journey [12, 13].

To measure an effective primary health care system, ambulatory-care-sensitive conditions have become a commonly used indicator [14]. Ambulatory-care-sensitive conditions are defined as conditions for which hospital admission can be prevented by early interventions in primary care [14]. Little data is available on ambulatory-care-sensitive conditions in asylum-seeking patients. An older study from Australia based on hospital discharge data between 1998 and 2004 found that admissions for ambulatory-care-sensitive conditions were lower among refugee-born individuals compared the resident population [15]. Two recent studies from Germany report contrasting findings. A study based on health care insurance data in children and adults including 3′639 asylum-seeking and 18′191 non-asylum-seeking individuals showed higher admission rates for ambulatory-care-sensitive conditions in asylum-seeking compared to non-asylum-seeking individuals [16].

Similarly, another study investigating ambulatory-care-sensitive conditions in over 32′000 pediatric patients in a single center emergency department in 2015 found a higher rate of asylum-seeking children admitted for ambulatory-care-sensitive conditions compared to non-asylum-seeking children [17].

Low integration in a primary health care system may result in delayed presentations leading to hospital admissions due to ambulatory-care-sensitive conditions. It may also result in increased numbers of presentations with non-urgent conditions at emergency departments. There is a global trend of an increase in non-urgent visits at emergency departments in high-income countries, which could also potentially be prevented by primary health care [18–21]. Asylum-seeking children are at risk of lacking integration into the primary health care system and may therefore have higher rates of ambulatory-care-sensitive admissions and non-urgent emergency-department presentations than their local peers.

The aim of this study was to assess the number of preventable hospital admissions and emergency department visits in asylum-seeking and non-asylum-seeking pediatric patients.

## Methods

### Study design

The study was a retrospective hospital-based study of the years 2016–2017, comparing asylum-seeking pediatric patients with non-asylum-seeking patients. The primary outcome was the proportion of admissions due to ambulatory-care-sensitive conditions in the asylum-seeking group compared to the non-asylum-seeking group. Secondary outcomes were the proportion of non-urgent visits in asylum-seeking outpatients presenting at the emergency department and the proportion of asylum-seeking children having an assigned primary care physician.

### Study setting

Located in Switzerland at the border to France and Germany, the University Children’s Hospital Basel delivers health care to a multicultural population. The hospital is part of the Swiss hospitals for equity program [22] and the only tertiary pediatric health care provider for two regions in North-West Switzerland. Basel has of the largest of the six Swiss reception centers for asylum-seeking individuals run by the Swiss State Secretary of Migration where asylum-seekers stay for a maximum of 3 months after arrival.

### Study population

Data of all visits at the University Children’s Hospital Basel was extracted from the administrative electronic health records from 1st Jan 2016 to 31st Dec 2017. For this analysis only visits of the emergency department and admissions were included. An admission was defined as a hospital visit including at least one overnight stay. To prevent an overestimation of visits, an emergency department contact which led to admission was counted as admission and marked as admission initiated by the emergency department but not counted as additional emergency department visit. Records showing visits of multiple departments during the same admission were counted as one admission. The asylum-seeking status is systematically assessed and recorded at this institution for all patients since 2016. Patients were registered as asylum-seeking if any of the following conditions were met: (i) referred from one of the reception centers run by the State Secretary for Migration; (ii) referral sheet stating that the patient is asylum-seeking; (iii) patient showing an asylum-seeking identity card, which is routinely issued to all individuals lodging an asylum request in Switzerland. Asylum-seeking children with visits recorded 1 year or longer before the study period (i.e. before 1st January 2015) were excluded from the current analysis, to ensure only recently arrived asylum-seeking patients were included in the analysis.

### Data collection

Data extraction for all identified patients was done using electronic administrative and medical health records for the following variables in all hospitalized patients: main diagnosis, asylum status, nationality, age, gender, duration of hospitalization, department hospitalized and admitting authority. For all visits of the asylum-seeking group, the following variables were manually extracted and added to the database: family structure, registered personal pediatrician, triage score, radiological exams and therapies prescribed. Extracted data was transferred to a Redcap-database (Vanderbilt University/IC 6.9.4). Data cleaning and automatic as well as manual quality control tests were performed. Automatic data cleaning was performed using validation rules for data entries as built-in checks for missing values, out of range data or outliers for numerical fields. In addition, data entry was manually checked by an independent person. The records were locked prior to analysis.

### Definition of ambulatory-care-sensitive conditions

Ambulatory-care-sensitive conditions were defined using criteria from previously published studies [23–29]. The final list defining ambulatory-care-sensitive conditions in our study was based on a recent study done in a context comparable to ours [17]. The list included 304 International Classification of Diseases-10 codes summarized in 17 categories (Supplementary material 1).

### Definition of non-urgent visits

To assess the proportion of non-urgent visits, the Australasian Triage Scale was used. The score was developed by the Australasian College for Emergency Medicine, revised in 2000, validated and is widely used [30]. It ranges from 1 (resuscitation) to 4 (less-urgent condition) and 5 (non-urgent condition). The score is routinely assessed by trained nurses in all patients presenting at the emergency department of the University Children’s Hospital Basel. Non-urgent visits were defined as triage score 4 or 5 as proposed in previous studies [31].

### Analysis

STATA (Stata/IC 13.12013) was used for the statistical analysis as for the generation of graphs. The statistical analysis was mainly descriptive. Inferential statistics were used to describe the primary outcome parameter. The two sample Chi-square test was used to compare proportions of the primary outcome parameter, namely the proportion of admissions due to ambulatory-care-sensitive conditions in the asylum-seeking group compared to the non-asylum-seeking group. Confidence intervals were provided to describe the precision around the summary statistic using a confidence level of 95%. To provide information about the completeness of the dataset, records with missing data were not excluded from analysis but reported as such.

### Ethics approval

The study was approved by the Ethics committee of North-West Switzerland (EKNZ 2017–01585).

## Results

### General

A total of 75′199 hospital visits were included, of which 63′405 were emergency department visits and 11′794 were admissions. Baseline Characteristics are summarized in Table 1:

**Table 1:** Baseline characteristics of emergency department visits and admissions by asylum-seeking and non-asylum-seeking patients in 2016–2017

**Characteristics**	**Visits by asylum-seeking patients**				**Visits by non-asylum-seeking patients**				
	**Admissions*n*** **= 149**		**Outpatient ED*n*** **= 297**		**Admissions*n*** **= 11′645**			**Outpatient ED*n*** **= 63′108**	
	**N**	**IQR/ %**	**N**	**IQR/%**	**N**		**IQR/%**	**N**	**IQR/%**
Median age	4	0–13	5	1–11	4		0–11	5	2–10
Male gender	88	59	175	59	6′456		55	34′416	55
Most frequent nationalities:									
Eritrea	34	23	48	16	Switzerland	7612	65	35′776	57
Afghanistan	26	17	40	13	Germany	916	8	4005	6
Syria	24	16	59	20	Italy	377	3	2475	4
Somalia	11	7	20	7	Turkey	363	3	3446	5
Iraq	9	6	17	6	Kosovo	331	3	2389	4
Missing data	1	1	4	1	Missing data	4	0	4	0
Other	44	30	109	37	Other	1991	17	15′013	24
Average days admitted	4	2–7	na	na	3		2–6	na	na
Admitted by					Admitted by				
ED	97	65	na	na	ED	7013	60	na	na
Referral	30	20	na	na	Referral	3223	28	na	na
Transfer other hospital	22	15	na	na	Transfer other hospital	1409	12	na	na
Missing	0	0							

*ED* Emergency department, *na* not applicable

**Fig. 1:**
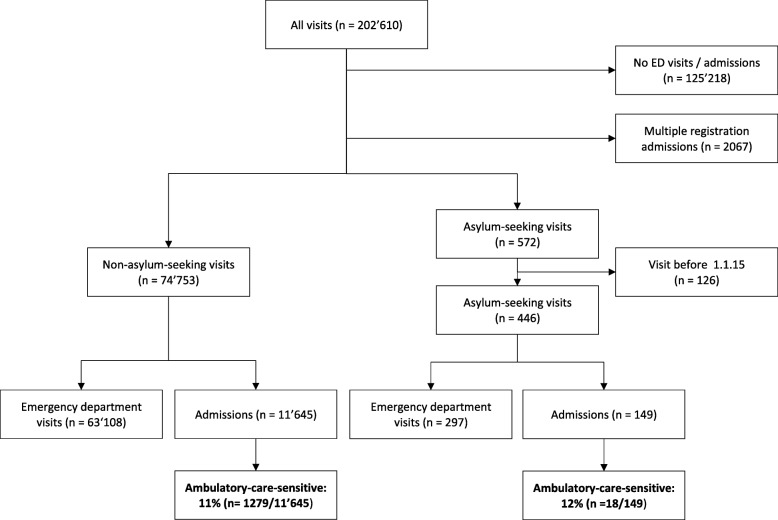
Flow-diagram showing the process of inclusion of the study population

Of the admissions, 149/11′794 (1.3%) were by asylum-seeking and 11′645/11′794 (98.7%) by non-asylum-seeking patients (Fig. 1). Admissions were mainly initiated by the emergency department in both groups: in 97/149 (65%) of the asylum-seeking and in 7013/11′645 (60%) of the non-asylum-seeking group. The remaining were admissions initiated by outpatient departments or planned admissions (Table 1).

### Admission for ambulatory-care-sensitive conditions

**Fig. 2:**
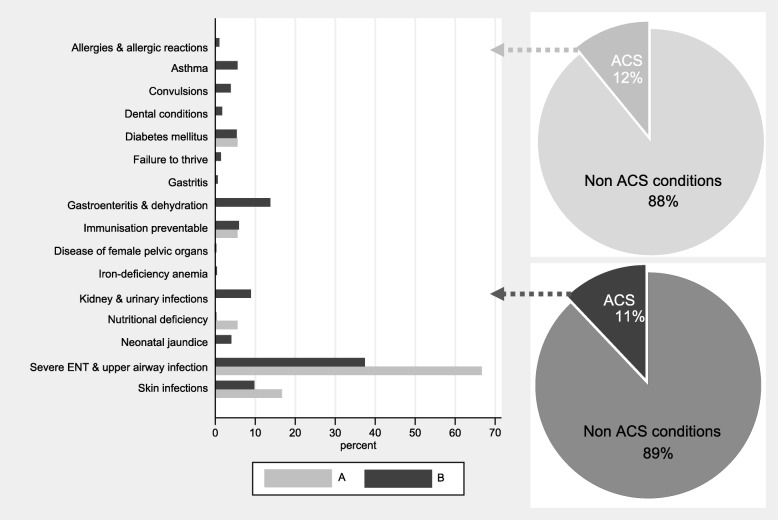
Bar-graph depicting the distribution and proportion of ambulatory-care-sensitive admissions in asylum-seeking (**A**) and non-asylum-seeking (**B**) patients. ACS = ambulatory-care sensitive conditions

Ambulatory-care-sensitive conditions accounted for 18/149 (12.1%; CI: 0.07–0.18) of the admissions in asylum-seeking and for 1270/11′645 (10.9%; CI: 0.1–0.11) in the non-asylum-seeking patients (*p* = 0.65; CI: − 0.04-0.06). The distribution within the different categories of ambulatory-care-sensitive conditions varied between the groups (Fig. 2). The most frequent category in both groups was “severe infections of ear, nose, throat or upper respiratory tract”. This ambulatory-care-sensitive condition category was more frequent in visits by asylum-seeking compared to non-asylum seeking patients, 12/18 (67%) and 475/1270 (37%) respectively. Skin infections were the second most common category in admissions for ambulatory-care-sensitive conditions by asylum-seeking patients with 3/18 (17%), compared to 124/1270 (10%) in non-asylum-seeking patients. In the non-asylum-seeking patients, admissions for “gastroenteritis and dehydration” was also common with 175/1270 (14%), compared to no admission for this reasons in the asylum-seeking patients. Nutritional deficiency was more common in asylum-seeking patients 1/18 (6%) compared to non-asylum-seeking patients 2/1270 (0%).

In both groups, the majority of admissions due to ambulatory-care-sensitive conditions were through the emergency department: 16/18 (89%) in the asylum-seeking and 1101/1270 (87%) in the non-asylum-seeking patients, respectively. Few patients were transferred from other hospitals: 1/18 (6%) in the asylum-seeking and 39/1270 (3%) or in the non-asylum-seeking patients; or referred by primary care physicians 1/18 (6%) in the asylum-seeking and 130/1270 (10%) in the non-asylum-seeking patients. A primary care physician was documented in 66/149 (44%) of the admissions of asylum-seeking patients. There was no difference in the proportion of admissions for ambulatory-care-sensitive conditions in visits with and without a documented primary care physician: 8/66 (12%) and 10/83 (12%), respectively.

### Emergency department visits and admissions through the emergency department

In total, 70′515 visits at the emergency department were recorded of which 63′405 were emergency department visits and 7′110 were admissions initiated by the emergency department. In total, 394/70′515 (0.5%) were by asylum-seeking and 70′121/70′515 (99.5%) by non-asylum-seeking patients. The proportion of emergency department contacts leading to admission was higher in asylum-seeking compared to non-asylum-seeking patients, 97/394 (25%) and 7013/70′121 (10%), respectively. In both groups, a large proportion of emergency department visits were by patients below 2 years of age: 115/394 (29%) in asylum-seeking and 15′126/70′121 (26%) in non-asylum-seeking patients.

### Details of emergency department visits in the asylum-seeking patients

Non-urgent visits were frequent in asylum-seeking patients with 82% (244/297) of the total visits. A primary care physician was documented in 122/297 (47%) of the asylum-seeking outpatient visits. The median (IQR) triages score for those with and without a documented primary care physician was similar: 5 (3–5) and 5 (4–5), respectively. The proportion of office-hours visits was similar in visits of patients with a primary care physician documented compared to visits of those without: 57% (70/122) versus 59% (99/168), respectively.

**Fig. 3:**
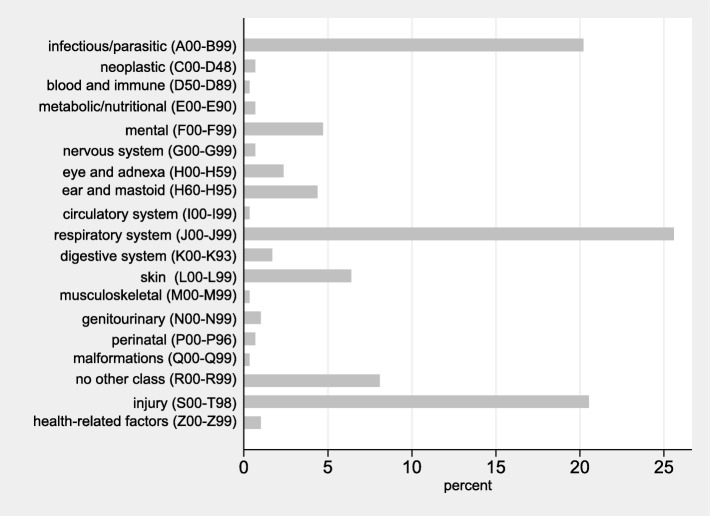
Bar-graph depicting the spectrum of disease (using ICD-10 coding) and proportion in emergency department outpatient visits in asylum-seeking patients. ICD = International classification of diseases

A detailed analysis of the spectrum of diseases in asylum-seeking patients with emergency department outpatient visits is shown in Fig. 3. A total of 165/297 (56%) of the visits in the asylum-seeking patients were due to an infectious disease, most commonly infection of the “respiratory system”. The most common single ICD-10 code was “Acute upper respiratory infections of multiple and unspecified sites” (J 06) with 54/297 (18%) visits. The second most frequent category was “injury” (S00-T98) with 61/297 (21%) visits, with most frequent single ICD-10 code being “superficial injury of head” (S00; 8/297; 3%) and “open wound of head” (S01; 7/297; 2%).

## Discussion

The current study is the largest study to-date which has systematically compared ambulatory-care-sensitive conditions in admissions of recent asylum-seeking and non-asylum-seeking pediatric patients. We found 11 to 12% overall of admissions to be ambulatory-care-sensitive conditions with no significant difference in the proportions in asylum-seeking and non-asylum-seeking pediatric patients. This finding contrasts results from two previous studies in Germany. One of these studies in a similar setting used the same ICD-10 codes to define ambulatory-care-sensitive conditions. It showed higher proportions of ambulatory-care-sensitive conditions leading to admission with 17% in the asylum-seeking compared to 10% in the non-asylum-seeking patients [17]. One potential reason for lower rates in our setting is the different local primary care system with nursing staff being present at reception center and cooperation with primary and tertiary care providers preventing admission for ambulatory-care-sensitive conditions. This is also shown by the fact that in half of the visits by asylum-seeking patients a primary care physician was documented in our setting. An alternative explanation for the difference between the two studies may be that children included in the study in Germany in 2015 were more likely admitted as local health care systems were overwhelmed by the high number of arriving asylum-seekers at that time [4, 32].

Of the few studies that reported ambulatory-care-sensitive conditions in asylum-seeking patients, an older Australian study reported similar admission rates to our study and also found no difference in rates between patients from refugee-source countries and residents [15]. Australia is a county with a long-standing immigrant health history and well-developed health systems for refugees and asylum-seekers. This fact may lead to similar levels of health care provided to asylum-seekers non asylum-seekers consequently reduces admissions for ambulatory-care-sensitive conditions. However these results may entail misclassification bias as the country of birth was used as a proxy to define the patient as refugee or resident.

In our study, the overall proportion of outpatient visits to the emergency department by asylum-seeking patients was low. This was confirmed in another study from the same institution that investigated all visits including other non-emergency-department outpatient visits showing that visits by asylum-seeking patients were less than 2% of the total visits [33]. A detailed analysis of the emergency department triage scores showed non-urgent visits to be frequent with over 80% in asylum-seeking children. This proportion is considerably higher than in previous reports of 30–40% of non-urgent visits in pediatric emergency care centers in Canada and Australia [19, 34], 40% in Belgium and Italy [35, 36] and 60% in the United States [37] . However, the tendency to provide primary care by tertiary health care institutions is described as global problem and one reason for growing health expenses [20]. In a study from the UK, no association between non-urgent presentations and the asylum-status was found [38]. Reasons for presenting to an emergency department rather than to a primary care physician have been investigated in a Canadian study and included high sense of urgency, the feeling of trust in the emergency health care workers and presence of equipment [19]. A potential solution may be anticipatory parent education as shown in a study in Minnesota where emergency department visits for ear pain were reduced by 80% after nurses provided preventive education and treatment for ear pain [39].

In our study, the spectrum of disease found in asylum-seeking outpatient visits in an emergency department was mainly consisting of respiratory tract infections and minor injuries of the head. Interestingly this is not different from non-asylum-seeking patients in high-income countries [40]. Differences between asylum-seeking and non-asylum seeking patients seem to be more pronounced in the way how health care is best delivered as asylum-seeking patients require particular attention to communication, confidence achieved by a trustful patient-provider relationship and continuity of care [6, 41].

Results from our study, which is the largest study to-date that systematically compared ambulatory-care-sensitive conditions in admissions of asylum-seeking and non-asylum-seeking pediatric patients, have several limitations. First, admissions due to ambulatory-care-sensitive conditions were relatively few in the asylum-seeking group, not allowing further stratification for sex and age. However, the risk of a significant influence is low as the sex and age distribution was comparable in both groups. Second the coding of ICD-10 codes might be subject to inter-individual variation of the coders. However, the coding of both groups was done by the same staff of the accounting unit of the hospital. Third, we were unable to analyze the frequency of non-urgent visits in the non-asylum seeking patients as this data was not available at the time analysis was performed.

## Conclusion

Admissions due to ambulatory-care-sensitive conditions were not significantly different in asylum-seeking and non-asylum-seeking children in a Swiss tertiary care pediatric hospital. This suggests a well-developed primary health-care system for asylum-seeking children in the local context. Non-urgent visits were frequent in asylum-seeking patients and new strategies are required to reduce this burden and improve cost-effectiveness of the current system.

## Supplementary information

**Additional file 1 Supplementary data 1:** Ambulatory-care-sensitive conditions categories and their codes adapted from Lichtl et al [17].

## Data Availability

The datasets used and/or analysed during the current study are available from the corresponding author on reasonable request.
