# Supplementary material for: Preventable admissions and emergency-department-visits in pediatric asylum-seeking and non-asylum-seeking patients
Source: Int J Equity Health. 2020 May 1;19:58. doi: 10.1186/s12939-020-01172-w (PMC7193367; doi:10.1186/s12939-020-01172-w)
Supplement: Supplementary file 1 — Additional file 1 Supplementary data 1: Ambulatory-care-sensitive conditions categories and their codes adapted from Lichtl et al [17]. [file 12939_2020_1172_MOESM1_ESM.docx]

**Supplementary data 1:** Ambulatory-care-sensitive conditions categories and their codes adapted from Lichtl et al ([17](#_ENREF_17))

| category | 1 | 2 | 3 | 4 |  | 5 | 6 | 7 | 8 | 9 |  |  | 10 | 11 | 12 | 13 | 14 | 15 | 16 | 17 |
| --- | --- | --- | --- | --- | --- | --- | --- | --- | --- | --- | --- | --- | --- | --- | --- | --- | --- | --- | --- | --- |
| Codes | J30.1  J30.2  J30.3  J30.4  K52.2  L23.0  L23.1  L23.2  L23.3  L23.4  L23.5  L23.6  L23.7  L23.8  L23.9  T78.4  Z88.0  Z88.1  Z88.2  Z88.3  Z88.4  Z88.5  Z88.6  Z88.7  Z88.8  Z88.9  Z91.0 | J45.0  J45.1  J45.8  J45.9 | R25.2  R56.8  Z82  Z86.6 | K00.2  K00.3  K00.4  K00.5  K00.6  K00.7  K00.8  K00.9  K01.0  K01.1  K02.0  K02.1  K02.2  K02.3  K02.4  K02.5  K02.8  K02.9  K03.0  K03.1  K03.2  K03.3  K03.4  K03.5  K03.6  K03.7  K03.8  K03.9 | K04.0  K04.1  K04.2  K04.3  K04.4  K04.5  K04.6  K04.7  K04.8  K04.9  K05.0  K05.1  K05.2  K05.3  K05.4  K05.5  K05.6  K06.0  K06.1  K06.2  K06.8  K06.9  K07.0  K07.1  K07.2  K07.3  K07.4  K07.8  K07.9 | E10.90  E10.91  E10.92  E10.93  E10.94  E10.95  E13.41  R73.0  R73.9 | E45  R62.8 | K29.0  K29.1  K29.2  K29.3  K29.4  K29.5  K29.6  K29.7  K29.8  K29.9 | A08.0  J10.8  J11.8 | A33  A35  A36.0  A36.1  A36.2  A36.3  A36.8  A36.9  A37.0  A37.1  A37.8  A37.9  A39.0  A39.1  A39.2  A39.3  A39.4  A39.5  A39.8  A39.9  A40.3  A80.0  A80.1  A80.2  A80.3  A80.4  A80.9  B01.0  B01.1  B01.2  B01.8  B01.9  B05.0 | B05.1  B05.2  B05.3  B05.4  B05.8  B05.9  B06.0  B06.8  B06.9  B15.9  B16.1  B16.9  B18.0  B18.1  B26.0  B26.1  B26.2  B26.3  B26.8  B26.9  G00.0  G00.1  I30.1  J14  J17.1  M00.10  M00.11  M00.12  M00.13  M00.14  M00.15  M00.16  M00.17  M00.18 | M00.19  P35.0  P35.8  Z22.2  Z22.5  Z23.0  Z23.1  Z23.2  Z23.3  Z23.4  Z23.5  Z23.6  Z23.7  Z23.8  Z24.0  Z24.1  Z24.2  Z24.3  Z24.4  Z24.5  Z24.6  Z25.0  Z25.1  Z25.8  Z26.0  Z26.8  Z26.9  Z27.0  27.1  Z27.2  Z27.3  Z27.4  Z27.8  Z27.9 | N70.0  N70.1  N70.9  N71.0  N71.1  N71.9  N72  N73.0  N73.1  N73.2  N73.3  N73.4  N73.5  N73.6  N73.8  N73.9  N74.0  N74.1  N74.2  N74.3  N74.4  N74.8 | D50.0  D50.1  D50.8  D50.9 | \| N15.11 \| \| --- \| \| N15.8 \| \| N15.9 \| \| N28.1 \| \| N28.8 \| \| N28.9 \| \| N39.0 \| \| N39.81 \| | E40  E41  E42  E43  E44.0  E44.1  E45  E46  E64 | P59.3  P59.8  P59.9 | H66.0  H66.1  H66.2  H66.3  H66.4  H66.9  J00  J01.0  J01.1  J01.2  J01.3  J01.4  J01.8  J01.9  J02.0  J02.8  J02.9  J03.0  J03.8  J03.9  J06.0  J06.8  J06.9  J31.0  J31.1  J31.2 | L03.01  L03.02  L03.10  L03.11  L03.2  L03.3  L03.8  L03.9  L04.0  L04.1  L04.2  L04.3  L04.8  L04.9 | Z91.1 |

Categories: 1) Allergies & allergic reactions, 2) Asthma, 3) Convulsions, 4) Dental conditions, 5) Diabetes mellitus, 6) Failure to thrive, 7) Gastritis, 8) Gastroenteritis / dehydration, 9) Immunization-preventable diseases, 10) Inflammatory diseases of female pelvic organs, 11) Iron deficiency anemia / anemia, 12) Kidney- and urinary infections, 13) Nutritional deficiency, 14) Neonatal jaundice, 15) Severe ENT & upper airway infection 16) Skin infection, 17) Doctor’s orders have not been followed by patient.
